# Association between Short-Term Exposure to Ozone and Heart Rate Variability: A Systematic Review and Meta-Analysis

**DOI:** 10.3390/ijerph191811186

**Published:** 2022-09-06

**Authors:** Zhiqiang Zong, Mengyue Zhang, Kexin Xu, Yunquan Zhang, Chengyang Hu

**Affiliations:** 1 Department of Clinical Medicine, The Second School of Clinical Medicine, Anhui Medical University, 81 Meishan Road, Hefei 230032, China; 2 School of Public Health, Wuhan University of Science and Technology, Wuhan 430065, China; 3 Department of Humanistic Medicine, School of Humanistic Medicine, Anhui Medical University, 81 Meishan Road, Hefei 230032, China; 4 Department of Epidemiology and Biostatistics, School of Public health, Anhui Medical University, 81 Meishan Road, Hefei 230032, China

**Keywords:** ozone, heart rate variability, systematic review, meta-analysis

## Abstract

At present, ambient air pollution poses a significant threat to patients with cardiovascular disease (CVD). The heart rate variability (HRV) is a marker of the cardiac autonomic nervous system, and it is related to air pollution and cardiovascular disease. There is, however, considerable disagreement in the literature regarding the association between ozone (O_3_) and HRV. To further investigate the effects of short-term exposure to O_3_ on HRV, we conducted the first meta-analysis of relevant studies. The percentage change of HRV indicator(s) is the effect estimate extracted for the quantitative analysis in this study. In our meta-analysis, per 10 ppb increase in O_3_ was significantly associated with decreases in the time-domain measurements, for standard deviation of the normal-to-normal (NN) interval (SDNN) −1.11% (95%CI: −1.35%, −0.87%) and for root mean square of successive differences (RMSSD) −3.26% (95%CI: −5.42%, −1.09%); in the frequency-domain measurements, for high frequency (HF) −3.01% (95%CI: −4.66%, −1.35%) and for low frequency (LF) −2.14% (95%CI: −3.83%, −0.45%). This study showed short-term exposure to O_3_ was associated with reduced HRV indicators in adults, which suggested that the cardiac autonomic nervous system might be affected after O_3_ exposure, contributing to the association between O_3_ exposure and CVD risk.

## 1. Introduction

The formation of ozone (O_3_) in the atmosphere is normally caused by the reaction between nitrogen oxides and volatile organic compounds under solar irradiation. At present, it is one of the most important pollutants associated with traffic in urban and industrialized areas and has been linked to a number of health outcomes, including cardiovascular diseases [1,2,3].

Globally, cardiovascular disease (CVD) is one of the leading causes of death, and its incidence is expected to rise steadily in the next decade [4]. Environmental air pollution has been estimated to be a major contributor to cardiovascular mortality worldwide [5], with a recent study reporting that cardiovascular disease was responsible for 18.6 million deaths in 2019 [6]. Nevertheless, there is still uncertainty as to whether short-term O_3_ exposure is causal and biologically responsible for higher cardiometabolic risks [7,8]. There has been recent research conducted on the impact of exposure to O_3_ on cardiovascular systems, but a consensus has not been reached due to varied reasons, such as study design, study population, or exposure measurement method [9,10]. In light of this, it is necessary to further investigate the impact of O_3_ exposure on cardiovascular health.

One method of predicting CVD risk is using surrogate markers, and heart rate variability (HRV) has been shown to be a reliable predictor. Specifically, an increase in HRV indicates that the autonomic nervous system (ANS) is well adapted and functioning efficiently, while a decrease in HRV is often an indication that the ANS has not been sufficiently adapted [11]. All HRV measures are calculated by recording and analyzing the interval between adjacent heartbeats, the inter beat interval (IBI in milliseconds). The most common method of measuring HRV is electrocardiography (ECG). The operationalization of HRV can be classified into two broad categories: time-domain and frequency-domain measures. Time-domain indices are derived directly from the R-R interval series and generally measure the variability contained therein by applying simple statistical computations, such as standard deviation of the normal-to-normal (NN) interval (SDNN) in milliseconds or log-transformed values, or root mean square of successive differences (RMSSD) between adjacent R-R intervals in milliseconds or log-transformed values [12]. Frequency-domain indices have been successfully used to evaluate the cardiac autonomic nervous system, of which high frequency (HF: 0.15–0.40 Hz) spectral power primarily reflects parasympathetic influences, whereas low frequency power (LF: 0.04–0.15 Hz) has been shown to reflect both sympathetic and parasympathetic influences [13,14].

Over the past several years, some studies have assessed the effects of short-term O_3_ exposure on HRV metrics; however, these results were inconsistent and a more comprehensive study is needed to elucidate the potential relationships [10,15,16]. In the meantime, the heterogeneity of the results across the epidemiologic literature warrants further investigation to better understand the underlying reasons contributing to these disparate findings in order to ultimately determine whether O_3_ exposure adversely affects the cardiovascular system.

To address this question of surrogate marker of CVD risk related to ozone exposure, we conducted a systematic review and meta-analysis of studies examining associations between short-term O_3_ exposure (measured on a continuous, rather than categorical, scale) and HRV metrics in the general population. This review uses the Population, Exposure, Comparator, Outcome, Study Design (PECOS) statement shown in Table 1.

## 2. Materials and Methods

The protocol of this study was not registered in PROSPERO.

### 2.1. Study Question

The search question was: “Among the general population, what is the effect of a higher exposure to ozone compared to lower level of ozone exposure on HRV indices?”.

### 2.2. Search Strategy

The PubMed, Embase, and Web of Science databases were searched for eligible studies between inception and 1 June 2022 using the following keywords, which are representative of the exposure and outcomes as described in our PECOS statement: (ozone or O_3_ or air pollution) AND (heart rate variability or HRV or root mean square of successive heartbeat interval differences or RMSSDs or standard deviation of NN intervals or SDNN) (Supplementary Materials). PRISMA (Preferred Reporting Items for Systematic Reviews and Meta-analyses) (Supplementary Materials) were followed in the reporting of this meta-analysis.

### 2.3. Study Selection

The eligibility criteria for the PECOS are summarized in Table 1. The study population was the general population. A 24-h average of ambient O_3_ exposure was used to correspond to personal exposure before the HRV protocol. The effect estimates (percentage change (%) and 95% confidence interval (CI)) in the indicators of HRV for an increase in O_3_ exposure by 10 ppb for a continuous exposure was considered. The outcome was HRV indicators, including RMSSD, SDNN, LF, and HF.

**Figure 1:**
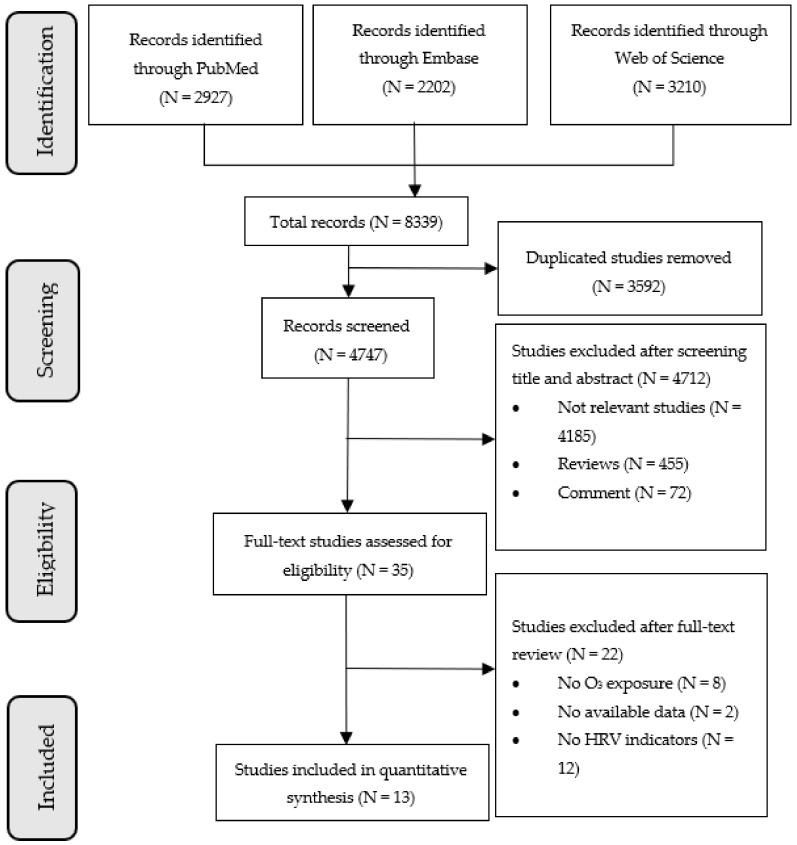
Flow chart of study selection.

Among the studies included in this review were cohort, case–control, and cross-sectional studies that examined the relationships between O_3_ and indicators of HRV published in English. We excluded conference papers, reviews, meta-analyses, and commentaries from the analysis. In order to be eligible for the analysis, studies had to be conducted in the general population and they had to contain original data providing effect estimates on at least one of these four HRV indicators: RMSSD, SDNN, LF, and HF. Those studies with overlapping populations and information were excluded from the review. In this case, we retained the publication providing the most complete information AND/OR the most representative population. Figure 1 shows the flow chart of the study selection process.

We selected studies following the screening of titles and abstracts by two investigators (Z.Q.Z. and M.Y.Z.), with any discrepancy being resolved by a third investigator (C.Y.H.); next, the retained potential eligible studies were screened on full-text reading.

### 2.4. Data Extraction and Quality Assessment

From each study, two investigators (Z.Z. and M.Z.) extracted the following data: author name, publication date, study country, study population, sample size, exposure assessment, indicator(s) of HRV, and adjusted covariates. For the purpose of gathering unpublished data, authors were contacted directly when it was considered appropriate. Meta-analysis was performed using the most fully adjusted effect estimate that represents the greatest control over potential confounders.

Cohort, panel, and case–crossover studies were assessed using Newcastle Ottawa Scale (NOS). There are eight items in the NOS, and the items are categorized into three dimensions, including selection, comparability, and outcomes. Studies were evaluated based on an NOS score from 0 to 9, with a score greater than 7 indicating high quality, a score between 5 and 6 indicating moderate quality, and one less than 5 indicating low quality [17].

### 2.5. Statistical Analysis

Meta-analyses were performed using fixed-effect or random-effects models for the associations of O_3_ exposure with four common indicators of HRV including two time-domain parameters (RMSSD and SDNN) and two frequency-domain parameters (LF and HF). As most of the included studies reported the HRV measurements on a logarithmic scale, we thus excluded the studies with linear scale models since they were not comparable. The effect estimates of each included study were presented per standard deviation (SD) or interquartile range (IQR) change of the O_3_ level, and they were converted into per 10 ppb increase in O_3_. We calculated the percentage change in accordance with the methodology of a previous meta-analysis that assessed the associations of PM_2.5_ exposure with HRV [18].

In order to determine publication bias, funnel plots and Egger’s regression tests were utilized, and a *p*-value of Egger’s test less than 0.05 was found to indicate the presence of publication bias. Additionally, the trim-and-fill method was used to evaluate the impact of publication bias when appropriate. Evaluation for presence of heterogeneity was carried out using (1) Cochran’s Q-test with *p*-value less than 0.05 signifying heterogeneity and (2) I^2^ statistics, where I^2^ greater than 50% indicated substantial heterogeneity [19]. The potential source(s) of heterogeneity between the studies was explored using subgroup analysis, based on the characteristics of the original studies and based on the possible influence factors. We performed leave-one-out analyses in order to identify potential outliers and influential studies as sensitivities. All the analyses were performed with Stata version 15.1 (Stata Corp, College Station, TX, USA).

## 3. Results

### 3.1. Characteristics of Included Studies

In total, 8339 records were retrieved from three electronic bibliographic databases. The flow chart shows the detailed screening process (Figure 1). A total of 13 studies were eventually included in our systematic review and meta-analysis, of which 10 were panel studies, 2 were case–crossover studies, and 1 was cohort study [20,21,22,23,24,25,26,27,28,29,30,31,32]. There were 8 studies conducted in North America and 5 in East Asia. The quality of all the included studies was assessed as moderate to high. Table 2 provides a more detailed overview of the included studies.

### 3.2. Association between O_3_ Exposure and HRV 

**Figure 2:**
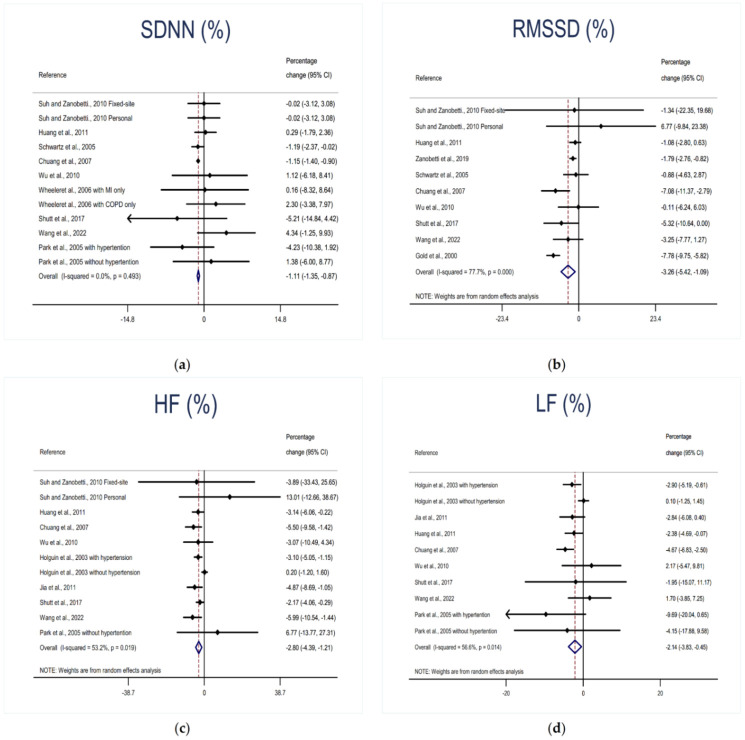
Forest plot of the meta-analysis: per 10 ppb increase in O_3_ exposure was associated with pooled percentage changes (%) in HRV indicators: (**a**) SDNN, (**b**) RMSSD, (**c**) HF, and (**d**) LF. MI: myocardial infarction; COPD: chronic obstructive pulmonary disease [20,21,22,23,24,25,26,27,28,29,30,31,32].

Thirteen studies have examined the relationships between short-term exposure to O_3_ and indicators of HRV. Per 10 ppb increase in O_3_ exposure was associated with a decrease in the indicators of HRV. Specifically, meta-analyses on associations of O_3_ exposure with RMSSD, HF, and LF showed moderate to high between-study heterogeneity and therefore a random-effects model was used, while for SDNN, a fixed-effects model was used due to the low between-study heterogeneity. As shown in Figure 2, the pooled estimates were −1.11% (95%CI: −1.35% to −0.87%, I^2^ = 0.0%) for SDNN and −3.26% (95%CI: −5.42% to −1.09%, I^2^ = 77.7%) for RMSSD, respectively. Similarly, the pooled estimates were −3.01% (95%CI: −4.66% to −1.35%, I^2^ = 59.7%) for HF and −2.14% (95%CI: −3.83% to −0.45%, I^2^ = 56.6%) for LF, respectively.

### 3.3. Subgroup Analysis

We performed the subgroup analyses by age of participants (≤35 year or ≥55 year), study location (North America or East Asia), length of ECG recording (≤30 min or others), O_3_ exposure (≤24 h or others), exposure assessment (fixed-site exposure or personal exposure), and quality of study (high or moderate) (Table 3). Subgroup analyses indicated that the effects of O_3_ exposure on the indicators of HRV in North America were more pronounced than in East Asia. For each assessed indicator of HRV, the associations seem inconsistent with each other by some stratified factors.

### 3.4. Sensitivity Analysis

**Figure 3:**
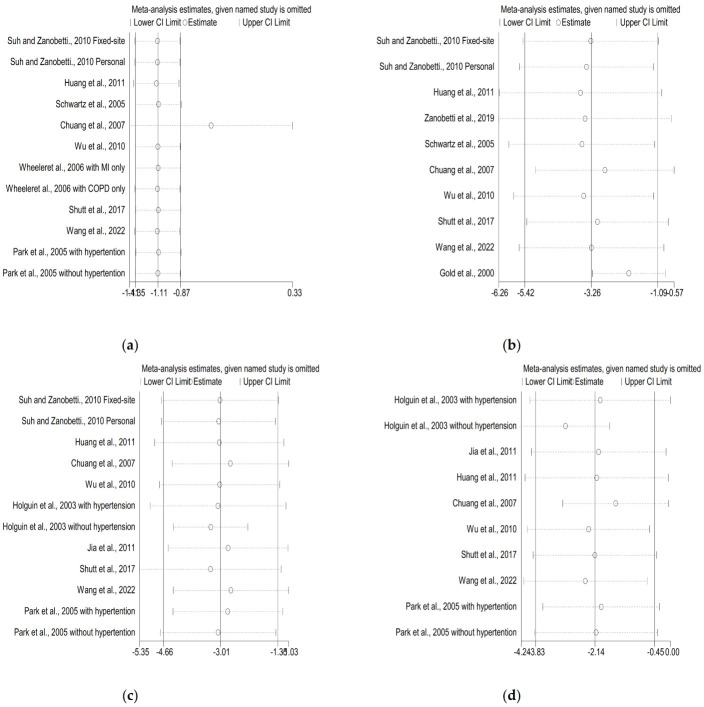
Sensitivity analysis of the association between short-term O_3_ exposure and HRV indicators: (**a**) SDNN, (**b**) RMSSD, (**c**) HF, and (**d**) LF [20,21,22,23,24,25,26,27,28,29,30,31,32].

Sensitivity analyses were performed to assess the stability of the results. Generally, the pooled estimates of O_3_ exposure on HRV indicators, such as RMSSD, HF, and LF, did not significantly change before and after systematically excluding each study, indicating the robustness of results (Figure 3). The indicator of SDDN, omitting one study at each time, showed that Chuang et al. 2007 was an influential study (Figure 3). When this study was excluded, we observed a non-significant association between O_3_ exposure and SDNN (−0.54%; 95%CI: −1.41% to 0.33, I^2^ = 0%).

### 3.5. Publication Bias

**Figure 4:**
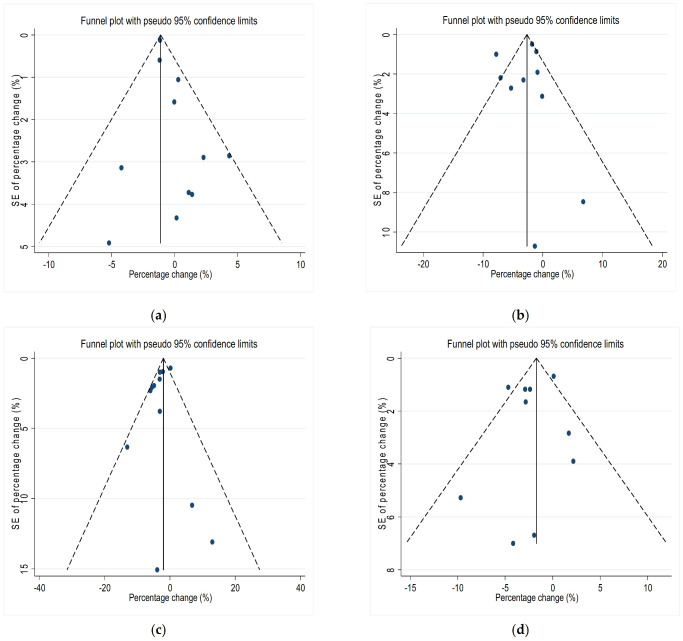
Funnel plot of the effects of short-term O_3_ exposure and HRV indicators. (**a**) SDNN (**b**) RMSSD (**c**) HF (**d**) LF. The ordinate axis in funnel plot represents standard error (SE) of percentage change (%).

We constructed vertical funnel plots and Egger’s tests to assess the publication bias for each O_3_ and HRV indicator combination. Vertical funnel plots showed basic symmetry (Figure 4). The *p* values for Egger’s tests were 0.090 for SDNN, 0.702 for RMSSD, 0.231 for HF, and 0.511 for LF, which indicates that there is no evidence of publication bias (*p* > 0.05).

## 4. Discussion

The purpose of this meta-analysis was to provide evidence that elevated levels of O_3_ can increase the risk of cardiovascular diseases in adults. In our meta-analysis, we evaluated the effects of O_3_ on HRV based on 13 observational studies conducted among the adults. According to the present meta-analysis, short-term exposure to O_3_ is associated with a decrease in HRV indices. The positive associations indicated that O_3_ may alter cardiac autonomic function and thus increase the risk of cardiovascular events. To the best of our knowledge, this is the most comprehensive meta-analysis which specifically evaluates the association between O_3_ exposure and HRV.

In general, the literature on the association between O_3_ exposure in the short-term and HRV is still scarce. The low number of studies included in the current meta-analysis, as well as the moderate to high heterogeneity observed in the study, may obscure the true association between O_3_ exposure and cardiovascular disease risk. Previous meta-analyses have shown that short-term exposure to O_3_ is associated with a variety of adverse health outcomes, including asthma exacerbations [33], pneumonia in children [34], pulmonary embolisms [35], and atrial fibrillation [36]. Despite this, no meta-analysis has been conducted on the connection between O_3_ exposure and cardiovascular disease. In the present meta-analysis, we emphasized the importance of integrating the results obtained from studies of people with cardiovascular disease with those obtained from studies of healthy individuals. The reason for this is that most previous studies have focused on older individuals with cardiopulmonary disease; there are only a small number of studies that examine associations among healthy and younger individuals. HRV indices have been shown to vary with cardiovascular status and drug mediation, and HRV responses to O_3_ stimulation are also thought to be affected by health status and drug mediation [37,38,39,40]. To gain a deeper understanding of the exposure–response association, evidence obtained from healthy people is essential.

The mechanisms by which exposure to O_3_ increases CVD risk have yet to be fully determined. It is a well-established fact that imbalance of ANS, as indicated by a disturbance of HRV, is one of the most important mechanisms by which O_3_ exposure increases the risk of adverse cardiovascular events [24,41]. An increase in LF/HF ratios and the withdrawal of parasympathetic nerves have been demonstrated as a key pathway in cardiovascular disease morbidity and mortality [42,43,44,45]. Furthermore, neuroendocrine stress responses have also been shown to contribute to cardiometabolic disease development. Hypothalamic–pituitary–adrenal (HPA) and sympathetic adrenal medullary (SAM) are essential components of neuroendocrine systems that maintain homeostasis in response to acute environmental stimuli [29,46]. High levels of O_3_ exposure may activate the HPA and SAM axis, triggering the release of stress hormones, such as corticotropin-releasing factor (CRF), adrenocorticotropic hormone (ACTH), cortisol, adrenaline, and noradrenaline, which further contribute to cardiovascular and metabolic dysfunction [47,48,49]. In addition, induction of oxidative stress and systemic inflammation are possible pathways through which O_3_ may affect the cardiovascular system. The initial responses to oxidant injury and inflammation may eventually result in endothelial dysfunction, acute arterial vasoconstriction, procoagulant activity, and atherosclerosis. The stimulation of nociceptive fibers in the airways may result in changes in sympathetic and/or parasympathetic tone, which may result in the onset of cardiac arrhythmias [16]. In general, autonomic dysfunction, neuroendocrine stress response, oxidative stress, and inflammation may be contributing factors to the increased cardiovascular risk associated with exposure to O_3_.

Moderate to high heterogeneity was detected in the meta-analysis and we further performed subgroup analyses. Several possible categorical variables were identified, such as age of participants, that could explain the heterogeneity among the combinations of O_3_ exposure with RMSSD and HF. We also observed a positive association between O_3_-HRV (significant percentage changes of SDNN, HF, and LF) in Asia, where levels of air pollution are much higher than North America [50]. From 2013 onwards, surface O_3_ levels have increased rapidly in China, during the warm season [51]. In the included studies, two methods of assessing O_3_ exposure were used, namely fixed-site monitoring and personal monitoring. The majority of studies on O_3_ exposure and cardiological diseases used measurements collected at centrally located monitoring stations or fixed-site; however, this method may introduce bias and distort epidemiological associations since it does not take into account the temporal variability of all possible sources of contamination and concentration. Moreover, most short-term effects studies of O_3_ used mean daily maximum 8-h average (MDA8) as exposure measurements and we only selected studies using 24-h averaged O_3_ to contain more comparable studies in the present study. Thus, the potential risk of exposure misclassification cannot be ruled out. In order to produce more accurate effect estimates, more detailed information on the measurements of various pollutants based on a fine spatiotemporal scale will provide more reliable understanding of the exposure–response associations [52]. Heterogeneity in the groups regarding ECG recording length and O_3_ exposure also exists. The record of electrocardiograms ranged from 5 min, 7 min, 15 min, 16 min, 30 min, 35 min, and 36 min to 24 h (ambulatory) in our included studies. However, a 5-min recording of electrocardiograms is recommended as longer recordings may be affected by emotions or physical activity [53]. The 24 h mean O_3_ concentration was the most commonly used; however, there were studies with O_3_ exposure periods of 1 h, 2 h, 4 h, 12 h, 24 h, 72 h and 120 h. Various mean period of O_3_ concentrations and ECG recording lengths do not produce the same effect estimates on HRV indicators, which may also explain the heterogeneity observed in the associations of O_3_ exposure with RMMSD, LF, and HF [18].

Furthermore, sensitivity analyses showed that our effect estimations for short-term O_3_ exposure and HRV indices were robust, with the exception of the combination of O_3_ and SDNN (Figure 3). The results for O_3_ and SDNN combination were not robust upon exclusion of this study (Chuang et al., 2007), suggesting that the mean period concentration of O_3_ of 72 h might have been the source of heterogeneity. Vertical funnel plots (Figure 4) and Egger’s tests indicated that there was no existence of publication bias among the assessed O_3_ and indicators of HRV combinations. Therefore, our study findings were reliable. Furthermore, the effects of O_3_ exposure on HRV indices might be explained by medication-induced modifications. For instance, in the study of Xing et al., air pollution exposure decreased 24-h SDNN by 1.31% (95%CI: 0.54−2.07%) in angiotensin receptor blocker (ARB) nonusers, whereas no obvious changes were observed in ARB users [54]. Peng et al. found that Diltiazem is more effective in treating stable coronary artery disease than ACEI/ARB and β-blockers [55]. Zhong et al. found that flavonoid intake with an increase in IQR was associated with a decrease of 5.09% (95%CI: 0.12−10.06%) in mean TLR2 methylation and prevented the negative effects of air pollution on LF [56]. As a result of the inclusion of different studies that have been adjusted for confounding factors, the pooled results may be heterogeneous. Several of the included studies ignored important confounding factors, including gender, BMI, temperature, humidity, season, and medication, and these factors may influence HRV. In light of this, future studies should take these perspectives into account.

This meta-analysis has several limitations that should be taken into account when interpreting the results. Firstly, there are a limited number of published studies available for each HRV indicator, which limits the statistical power of the analysis. Secondly, the studies included in the present study were observational, so we were unable to determine whether or not there was a causal relationship. Thirdly, the meta-analysis was based on studies conducted in North America and Asia, which limited the generalization of the results to the different geographical regions. Despite the aforementioned limitations, our meta-analysis has several strengths. As far as we are aware, this is the first meta-analysis conducted to examine the relationships between O_3_ exposure and HRV indices in the general population. We were able to perform multiple subgroup analyses to investigate the source(s) of heterogeneity. Finally, this meta-analysis included older adults with CVD as well as healthy young adults, which contributes to our understanding of the exposure–response relationship between O_3_ and HRV.

## 5. Conclusions

In this systematic review and meta-analysis, there is evidence that short-term exposure to O_3_ is associated with alterations in cardiac autonomic function, as measured by HRV in the general population. Further research is recommended to determine effective interventions for improving air quality and reducing incident CVD, and mechanistic studies are needed to determine the cause of the detrimental effects of ozone on the cardiovascular system.

## Figures and Tables

**Table 1:** PECOS for epidemiology study identification.

PECOS Element	Evidence
Population	General population, of all ages, developed and developing areas, both urban and rural. No geographical restrictions.
Exposure	Exposure to ambient O_3_ pollution. Exposure was expressed in continuous.
Comparator	A comparation population exposed to lower levels of O_3_ pollution.
Outcomes	Heart rate variability including four common indicators (RMSSD, SDNN, LF, and HF).
Study design	Cohort, nested or not nested case–control, case–cohort, or cross-sectional study designs, were considered.

**Table 2:** Basic characteristics of the studies included in the meta-analysis.

Author and Year of Publication	Study Location, Period and Design	Study Population	Outcome Assessment	Ozone Exposure Time	Monitoring Type	Adjusted Covariates	Heart Rate Variability Indicators and Percentage Change (%)	NOS Score
Suh and Zanobetti, 2010 [28]	Atlanta (USA), Fall 1999 and Spring 2000 Panel study	30 subjects: 12 with a recent myocardial infarction and 18 with chronic obstructive pulmonary disease Mean age: 65 year, 57% male	min ECG daily on seven consecutive days in one or both seasons. The ECG protocol involved 5 min of rest, 5 min of standing, 5 min of exercise out- doors, 5 min of recovery, and 20 cycles of slow breathing	24 h	Fixed-site; Personal exposure	Body mass index (BMI), temperature, relative humidity, sex, age, season, hour of day, day of week, medications use (beta-blockers, calcium channel blockers, angiotensin converting enzyme (ACE) inhibitors, and bronchodilators)	Per 16.02 ppb increase: SDNN: −0.03 (−8.40, 9.10) RMSSD: 10.83 (−12.63, 40.58) HF: 20.84 (−13.47, 68.76)	8
Huang et al., 2011 [23]	Beijing (China), during summer 2007 and summer 2008 Panel study	40 nonsmoking CVD patients (mean age = 65.6 years (standard deviation, 5.8) recruited through the on-campus clinic of Peking University Health Science Center (PKUHSC. A subset of 23 patients participated in 24-h ambulatory blood pressure monitoring	Consecutive 5-min measurements of heart rate and various measures of HRV were calculated for each monitoring session of each subject using personal computer-based software	12 h	Fixed-site	Age, BMI, gender, time of day, day of the week, visit, temperature, and relative humidity	Per 27.7 ppb increase SDNN: 0.8 (−1.8, 3.5) RMSSD: −3.0 (−7.6, 1.9) HF: −8.7 (−16.4, −0.2) LF: −6.6 (−12.8, −0.01)	8
Zanobetti et al., 2010 [32]	Boston (USA), 1999–2003 Panel study	46 patients with coronary artery disease, mean age: 57 year, 80% male, non-smoking	24 h ambulatory ECG, up to four with approximately 3-month intervals between visits	120 h	Fixed-site	Day of the week, traffic, average heart rate, hour of the day, date, mean temperature	Per 19 ppb increase RMSSD: −3.4 (−5.2, −1.5)	8
Wheeler et al., 2006 [30]	Atlanta (USA), Fall 1999 and Spring 2000 Panel study	30 subjects: 12 with a recent myocardial infarction and 18 with chronic obstructive pulmonary disease Mean age: 65 year, 57% male	min ECG daily on seven consecutive days in one or both seasons The ECG protocol involved 5 min of rest, 5 min of standing, 5 min of exercise out- doors, 5 min of recovery, and 20 cycles of slow breathing	4 h	Fixed-site	BMI, temperature, relative humidity, sex, age, season, hour of day, day of week, medications use (beta-blockers, calcium channel blockers, angiotensin converting enzyme (ACE) inhibitors, and bronchodilators)	Total (per 9.61 ppb increase) SDNN: 0.75 (−3.6, 5.3) With MI (per 8.08 ppb increase) SDNN: 0.13 (−6.5, 7.2) With COPD (per 10.66 increase) SDNN: 2.45 (−3.4, 8.7)	7
Schwartz et al., 2005 [26]	Boston (USA), Summer 1999 Panel study	28 subjects living near the exposure and health monitoring site, 61–89 year, 25% male myocardial infarction (n = 3), congestive heart failure (n = 2), chronic pulmonary disease (n = 2)	30-min ECG weekly over 12 weeks The ECG protocol involved 5 min of rest, 5 min of standing, 5 min of exercise outdoors, 5 min of recovery, and 3 min and 20 s of slow breathing	24 h	Fixed-site	Temperature, day of the week, hour of the day, medication use, time trend	Per 26 ppb increase SDNN: −1.5 (−5.7, 2.9) RMSSD: −2.3 (−11.6, 7.9)	6
Holguin et al., 2003 [22]	Mexico City (Mexico), 8 February–30 April 2000 Panel study	34 elderly residents of a nursing home, hypertension (n = 13), diabetes mellitus (n = 6), Parkinson’s disease (n = 4), chronic bronchitis (n = 4), 60–96 year, 44% male	5-min resting ECG in supine position, every other day be- tween 8:00 a.m. and 1:00 p.m. for three months	1 h	Fixed-site	Age, heart rate	Per 10 ppb increase Total HF: −0.1 (−0.016, 0.013) LF: −0.5 (−0.019, 0.009) With hypertension HF: −1.4 (−4.0, 1.2) LF: −2.1 (−0.045, 0.003) Without hypertension HF: 0.007 (−0.010, 0.024) LF: 0.005 (−0.011, 0.022)	6
Jia et al., 2011 [24]	Beijing (China), Summer 2008 and Winter 2009 Panel study	20 healthy elderlies, mean age 58.7 year, living near busy road, 25% male, non-smoking	Two 24 h ambulatory ECGs: one in summer 2008; one in winter 2009	2 h	Fixed-site	PM_2.5_, NO_x_, temperature, relative humidity, gender, age, BMI, survey number, activity	Per 10 ppb increase HF: −4.87 (−8.62, −0.97) LF: −2.84 (−6.03, 0.46)	7
Chuang et al., 2007 [20]	Taipei (China), April–June of 2004 or 2005 Panel study	76 healthy college students, no history of cardiovascular disease and of smoking, mean age: 21 year, 60% male	One monthly 16 min resting ECG in the sitting position, during daytime (8 a.m. to 2 p.m.), for three months (~30 days between measurements)	72 h	Fixed-site	Sex, age, BMI, weekday, temperature of day before, relative humidity	Per 12.0 ppb increase SDNN: −8.3 (−10.1, −6.5) RMSSD: −8.5 (−13.6, −3.3) HF: −6.6 (−11.8, −1.4) LF: −5.6 (−8.2, −3.0)	6
Wu et al., 2010 [31]	Taipei (China), February–March 2007 Panel study	17 healthy mail carriers, 32.4 year, 100% male, non-smoking	Ambulatory electrocardiographic data were collected continuously during their working periods, starting and ending 30 min before and after the mail delivery periods	24 h	Personal exposure	Age, BMI, second-hand smoke exposure, temperature during the working period	Per 17.6 ppb increase SDNN: 1.97 (−10.06, 15.62) RMSSD: −0.19 (−10.40, 11.19) HF: 5.41 (−7.60, 20.25) LF: 3.82 (−8.76, 18.13)	6
Shutt et al., 2017 [27]	Ottawa (Canada), Summer 2010 Case–crossover study	60 healthy adults, 24.2 ± 5.8 year, 46 male, 14 female	HRV analysis was undertaken on a segment of the ambulatory ECG recording during a 15 min rest period, near the end of the 8-h on-site day	120 h	Fixed-site	Age, heart rate, sex, BMI, temperature and relative humidity	Per 8.7 ppb increase SDNN: −5.59 (−10.01, 1.18) RMSSD: −6.11 (−10.87, 1.36) HF: −2.50 (−4.67, −0.33) LF: −2.24 (−17.32, 12.84)	7
Wang et al., 2022 [29]	Shanghai (China) October to November 2018 Case–crossover study	22 young participants (10 males and 12 females, 18–30 year) with complete data for final analyses	24-h ECG monitoring was performed using a 3-lead electrographic Holter monitor (Seer Light, GE Medical Systems) with a sampling rate of 128 Hz	2 h	Fixed-site	Age, sex, BMI, the collinearity between ozone and relative humidity in chamber	Per 10 ppb increase SDNN: 4.34 (−1.15, 10.14) RMSSD: −3.25 (−7.66, 1.38) HF: −5.99 (−10.44, −1.33) LF: 1.7 (−3.71, 7.40)	8
Gold et al., 2000 [21]	Boston (USA) May to July 1997 Panel study	21 volunteers, 73.3 year, 10 males and 11 females	25 min per week of continuous ECG monitoring, including 5 min of rest, 5 min of standing, 5 min of exercise outdoors, and 5 min of recovery	1 h	Fixed-site	Age, BMI, sex, smoking status, race, medication use, hypertension, coronary artery disease (history of angina or heart attack), history of congestive heart failure	Per 23.0 ppb increase RMSSD: −17.9 (−7.66, 1.38)	6
Park et al., 2005 [25]	Boston (USA) 14 November 2000–30 October 2003 Cohort study	497 elderly men, 72.7 ± 6.6	After the participants had rested for 5 min, the ECG was recorded for approximately 7 min with the subject seated. The best 4-consecutive-minute interval was used for the HRV calculations	4 h	Fixed-site	Age, BMI, mean arterial blood pressure (MAP), fasting blood glucose (FBG), cigarette smoking, use of beta-blocker, calcium-channel blocker, and/or ACE inhibitor, room temperature, season, and cubic smoothing splines (3 df) for moving averages of apparent temperature corresponding for the predictor	Per 13.0 ppb increase With hypertension SDNN: −5.5 (−15.7, 0.3) HF: −17.0 (−31.6, 0.7) LF: −12.6 (−25.0, 1.9) Without hypertension SDNN: 1.8 (−7.4, 11.8) HF: 8.8 (−14.7, 38.7) LF: −5.4 (−21.6, 14.1)	7

**Table 3:** Subgroup analysis of percentage change in indicators of HRV in association with each 10 ppb increase in short-term O_3_ exposure.

HRV Indices	Subgroup	Subgroup Criteria	Pooled Percentage Changes (%) with 95%CI	No. of Effect Estimates	No. of Studies	Heterogeneity	
						I^2^ (%)	*p* Value for Heterogeneity
SDNN	Age of participants	≤35 year	−0.15 (−3.09, 2.79)	4	4	36.8	0.191
		≥55 year	−0.65 (−1.54, 0.24)	8	5	0.0	0.710
	Study location	North America	−0.91 (−1.89, 0.08)	8	5	0.0	0.733
		East Asia	−1.12 (−1.37, −0.87)	4	4	48.8	0.119
	ECG recording length	Length of ECG ≤ 30 min	−0.89 (−1.88, 0.09)	5	3	0.0	0.541
		Others	−1.12 (−1.37, −0.87)	6	4	19.0	0.290
	O_3_ exposure time	O_3_ exposure < 24 h	−0.90 (−0.90, 2.70)	4	3	0.0	0.563
		Others	−1.14 (−1.39, −0.90)	8	6	0.0	0.863
	Exposure assessment	Fixed-site exposure	−1.12 (−1.36, −0.87)	10	8	6.2	0.385
		Personal exposure	−0.16 (−2.70, 3.01)	2	2	0.0	0.778
	Quality of study	High	−0.23 (−1.09, 1.55)	9	6	0.0	0.650
		Medium	−1.15 (−1.40, −0.91)	3	3	0.0	0.828
RMSSD	Age of participants	≤35 year	−4.36 (−7.13, −1.59)	4	4	19.9	0.290
		≥55 year	−2.67 (−5.55, 0.21)	6	5	85.4	<0.001
	Study location	North America	−3.43 (−7.02, 0.16)	6	5	84.3	<0.001
		East Asia	−2.81 (−5.78, 0.17)	4	4	58.0	0.067
	ECG recording length	Length of ECG ≤ 30 min	−3.78 (−8.20, 0.67)	4	4	88.9	<0.001
		Others	−2.52 (−4.50, −0.54)	6	5	31.3	0.201
	O_3_ exposure time	O_3_ exposure < 24 h	−4.08 (−9.01, 0.85)	3	3	92.1	<0.001
		Others	−2.55 (−4.56, −0.54)	7	6	32.1	0.183
	Exposure assessment	Fixed-site exposure	−3.69 (−5.98, −1.39)	8	8	81.8	<0.001
		Personal exposure	−0.72 (−5.04, 6.47)	2	2	0.0	0.446
	Quality of study	High	−1.74 (−2.56, −0.92)	6	5	0.0	0.586
		Medium	−4.38 (−8.42, −0.33)	4	4	78.7	0.003
HF	Age of participants	≤35 year	−3.56 (−5.61, −1.51)	4	4	20.9	0.285
		≥55 year	−2.54 (−4.90, −0.17)	8	5	62.1	0.014
	Study location	North America	−1.75 (−3.89, 0.39)	7	4	56.4	0.032
		East Asia	−4.11 (−6.20, −2.62)	5	5	0.0	0.802
	ECG recording length	Length of ECG ≤ 30 min	−2.10 (−3.88, −0.32)	7	5	57.4	0.029
		Others	−5.22 (−7.58, −2.86)	5	4	0.0	0.716
	O_3_ exposure time	O_3_ exposure < 24 h	−2.92 (−5.23, −0.62)	5	4	75.1	0.003
		Others	−3.28 (−5.75, −0.81)	7	5	14.6	0.318
	Exposure assessment	Fixed-site exposure	−3.10 (−4.83, −1.37)	10	8	60.9	0.006
		Personal exposure	0.08 (−12.44, 12.60)	2	2	28.2	0.238
	Quality of study	High	−3.42 (−5.15, −1.68)	8	6	14.3	0.318
		Medium	−2.43 (−5.20, 0.34)	4	3	74.9	0.007
LF	Age of participants	≤35 year	−1.33 (−5.70, 3.03)	4	4	54.8	0.084
		≥55 year	−2.02 (−3.80, −0.25)	6	4	51.9	0.065
	Study location	North America	−1.86 (−4.51, 0.78)	5	5	50.1	0.091
		East Asia	−2.50 (−4.52, −0.49)	5	5	43.4	0.133
	ECG recording length	Length of ECG ≤ 30 min	−1.62 (−3.43, 0.19)	7	5	40.8	0.119
		Others	−2.79 (−5.77, 0.19)	3	3	56.8	0.099
	O_3_ exposure time	O_3_ exposure < 24 h	−1.49 (−3.14, 0.16)	5	4	53.8	0.070
		Others	−4.29 (−6.37, −2.20)	5	4	0.8	0.402
	Exposure assessment	Fixed-site exposure	−2.33 (−4.07, −0.58)	9	7	59.4	0.011
		Personal exposure	--	--	--	--	--
	Quality of study	High	−2.34 (−4.07, −0.62)	6	5	81.2	0.001
		Medium	−1.94 (−4.76, 0.87)	4	4	0.0	0.530

## Data Availability

The data presented in this study are available on request from the corresponding authors.

## Supplementary Materials

The following supporting information can be downloaded at: https://www.mdpi.com/article/10.3390/ijerph191811186/s1, Table S1: Database search term list; Table S2: Preferred Reporting Items for Systematic reviews and Meta-Analysis (PRISMA) 2009 Checklist; Table S3: Quality evaluation using Newcastle Ottawa Scale (NOS) for 13 observational studies included in the meta-analysis.
